# Tick-Borne Encephalitis Virus Sequenced Directly from Questing and Blood-Feeding Ticks Reveals Quasispecies Variance

**DOI:** 10.1371/journal.pone.0103264

**Published:** 2014-07-24

**Authors:** Naveed Asghar, Pontus Lindblom, Wessam Melik, Richard Lindqvist, Mats Haglund, Pia Forsberg, Anna K. Överby, Åshild Andreassen, Per-Eric Lindgren, Magnus Johansson

**Affiliations:** 1 School of Natural Science, Technology & Environmental Studies, Södertörn University, Huddinge, Sweden; 2 Division of Medical Microbiology, Department of Clinical and Experimental Medicine, Linköping University, Linköping, Sweden; 3 Department of Clinical Microbiology, Virology, Umeå University, Umeå, Sweden; 4 Department of Infectious Diseases, County Hospital, Kalmar, Sweden; 5 Division of Infectious Diseases, Department of Clinical and Experimental Medicine, Linköping University, Linköping, Sweden; 6 Clinic of Infectious Diseases, Linköping University Hospital, Linköping, Sweden; 7 Division of Infectious Disease Control, Department of Virology, Norwegian Institute of Public Health, Oslo, Norway; 8 Division of Medical Services, Department of Microbiology, County Hospital Ryhov, Jönköping, Sweden; 9 School of Medicine, Örebro University, Örebro, Sweden; 10 iRiSC - Inflammatory Response and Infection Susceptibility Centre, Faculty of Medicine and Health, Örebro University, Örebro, Sweden; University of Hyderabad, India

## Abstract

The increased distribution of the tick-borne encephalitis virus (TBEV) in Scandinavia highlights the importance of characterizing novel sequences within the natural foci. In this study, two TBEV strains: the Norwegian Mandal 2009 (questing nymphs pool) and the Swedish Saringe 2009 (blood-fed nymph) were sequenced and phylogenetically characterized. Interestingly, the sequence of Mandal 2009 revealed the shorter form of the TBEV genome, similar to the highly virulent Hypr strain, within the 3′ non-coding region (3′NCR). A different genomic structure was found in the 3′NCR of Saringe 2009, as in-depth analysis demonstrated TBEV variants with different lengths within the poly(A) tract. This shows that TBEV quasispecies exists in nature and indicates a putative shift in the quasispecies pool when the virus switches between invertebrate and vertebrate environments. This prompted us to further sequence and analyze the 3′NCRs of additional Scandinavian TBEV strains and control strains, Hypr and Neudoerfl. Toro 2003 and Habo 2011 contained mainly a short (A)3C(A)6 poly(A) tract. A similar pattern was observed for the human TBEV isolates 1993/783 and 1991/4944; however, one clone of 1991/4944 contained an (A)3C(A)11 poly(A) sequence, demonstrating that quasispecies with longer poly(A) could be present in human isolates. Neudoerfl has previously been reported to contain a poly(A) region, but to our surprise the re-sequenced genome contained two major quasispecies variants, both lacking the poly(A) tract. We speculate that the observed differences are important factors for the understanding of virulence, spread, and control of the TBEV.

## Introduction

The tick-borne encephalitis virus (TBEV) is a human pathogen causing severe encephalitis across large parts of Europe and Asia. Three genetically distinct subtypes of TBEV, which are named after their geographical distribution include Western European- (W-), Far Eastern- (FE-), and Siberian- (S-) TBEV [1]. The natural life cycle of TBEV primarily involves zoonotic cycles between ticks and rodent hosts [2], [3]. Both the prevalence and incidence of TBE have increased over the last few decades [4], [5], which calls for thorough epidemiological and clinical investigations. TBEV contains an ∼11 kb positive sense, single-stranded RNA genome encoding a single polyprotein, flanked by the 5′- and 3′- non-coding regions (NCRs). The polyprotein is processed into three structural proteins: capsid (C), membrane (prM), and envelope (E), and seven non-structural (NS) proteins: NS1, NS2A, NS2B, NS3, NS4A, NS4B, and NS5 [6], [7]. The 5′NCR is highly conserved, whereas the 3′NCR can be divided into a conserved core element (C 3′NCR) and a variable part (V 3′NCR) [8], [9]. The V 3′NCR of the TBEV is heterogenic between the strains, both in nucleotide sequence and in length [9]. The longest W-TBEV genome detected so far is the virus of strain Neudoerfl, containing an internal poly(A) sequence with varying lengths 30–250 nt; however, the role of the heterogenic poly(A) tract in the viral life cycle is still unclear and has been suggested to be the product of laboratory virus cultivation [8]–[10]. Several W-TBEV strains have a similar genomic sequence as Neudoerfl besides the poly(A) being replaced by a homogenous (A)3C(A)6 sequence, e.g., in the Scandinavian strain Toro 2003. In addition, a number of W-TBEV strains lack the poly(A) tract and different truncations of the V 3′NCR exist, where the highly virulent strain Hypr represents the shortest W-TBEV strain sequenced [9]. The most available TBEV sequences have been obtained from the virus strains cultivated in suckling-mouse brain or cell culture; moreover, it has been shown that these cultivations can result in spontaneous genomic deletions within the V 3′NCR [11], [12].

Even though the clusters of virus variants have been shown to exist within the TBEV pool [13], little is known about the importance and existence of quasispecies within the natural life cycle of the TBEV. In addition, the increased distribution of the TBEV in Scandinavia highlights the importance of characterizing novel virus genomes and the genomic structures present within the natural foci. Here, we present two genomic sequences of the TBEV strains, Mandal 2009 and Saringe 2009, sequenced from the total RNA extract of questing and blood-feeding *Ixodes ricinus*, respectively. The TBEV of the blood-fed tick (Saringe 2009) had a heterogenic genomic section within the 3′NCR at the poly(A), reflecting a putative shift in the quasispecies pool when the viral replication switches from invertebrate to vertebrate hosts. This prompted us to further sequence and analyze the 3′NCRs of additional Scandinavian TBEV strains. The presented divergence could be key factors for the strain-dependent differences in the virulence observed for W-TBEV.

## Materials and Methods

### TBEV strains

The eight TBEV isolates investigated in this paper are listed in Table 1. Two TBEV isolates came from pools of questing ticks: Mandal 2009, from a pool of 10 questing nymphs sampled from Mandal, Norway (58°0.43' N, 7°30' E) and Toro 2003, from a pool of 106 questing nymphs and 9 questing adults sampled at Toro, Sweden (60°0.38′ N, 18°3.3′ E). Two TBEV isolates were obtained directly from the *I. ricinus* ticks detached from humans: Saringe 2009, from an engorged nymph after >60 h of blood-feeding on a 72-year-old male bitten in Saringe, Sweden (60°0.38′ N, 18°3.3′ E) and the strain Habo 2011, from a nymph after 12–24 h of blood-feeding on a 68-year-old male bitten in Habo, Sweden (57°54.9' N, 14°6.3' E). Both the Saringe and the Habo ticks were collected within the STING-study where the study participants gave their written informed consent before entering the study. Ethical approval for the TBD STING-study was granted by the Regional Ethics Committee in Linköping (M132-06), following the principles expressed in the declaration of Helsinki. Two TBEV isolates, 1991/4944 and 1993/783, were recovered from the blood of two Swedish patients during the first phase of the TBE disease. We also included two reference strains: Neudoerfl, isolated in 1971 from an *I. ricinus* tick in Burgenland, Austria and Hypr, isolated in 1953 from the blood of a deceased child in Moravia, Czech Republic.

**Table 1:** TBEV isolates included in the characterization of 3′ non-coding region.

Strain	Isolation-source^a^	Country	Isolation year	Reference
Mandal 2009	Questing *I. ricinus* (pool of 10N)	Norway	2009	(16)
Toro 2003	Questing *I. ricinus* (pool of 9A+106N)	Sweden	2003	(20)
Saringe 2009	>60 h blood-feeding *I. ricinus* on human (1N)	Sweden	2009	(17)
Habo 2011	12–24 h blood-feeding *I. ricinus* on human (1N)	Sweden	2011	This study
1991/4944	Blood from TBE patient during the first phase^b^	Sweden	1991	(14)
1993/783	Blood from TBE patient during the first phase^b^	Sweden	1993	(14)
Neudoerfl	*I. ricinus* c	Austria	1971	(9)
Hypr	Blood from child deceased in TBE^c^	Czech Republic	1953	(9)

a N: Nymph, A: Adult

b Initial isolation in suckling baby mice and then propagated on VeroB4 cells.

c Passaged an unknown number of times by inoculation of suckling baby mice.

### RNA isolation and cDNA synthesis

Ticks were homogenized for 2 min at 25 Hz in 450 µL RLT-buffer (Qiagen), supplemented with 1% β-mercaptoethanol using 5 mm steal bead (Eppendorf AG, Hamburg, Germany). The homogenized ticks were centrifuged at 20,000 g for 3 min followed by RNA extraction from 400 µL of the supernatant using Magattract RNA Tissue Mini M48 kit (Qiagen) in a Biorobot M48 workstation (Qiagen). The TBE patient sera were intracerebrally inoculated into two-day-old mice followed by viral isolation of the TBEV strains 1991/4944 and 1993/783, (passage 1) [14]. Viral stocks of 1991/4944 and 1993/783 were propagated in the VeroB4 cells (passage 2), and a total RNA isolation for sequencing was performed on the virus-infected VeroB4 cells (passage 3) using NucleoSpin RNA II (Macherey Nagel) according to the manufacturer's instructions. HEK 293T cells were infected (MOI of 1) with the Neudoerfl or Hypr strains (both with unknown passage history), and the total RNA was extracted 24 h post infection using NucleoSpin RNA II as described above. TBEV cultivation was done in the biosafety level 3 facility at Department of Clinical Microbiology, Umeå University.

RNA was mixed with dNTP mix and 10 pmol 3′NCR specific reverse primer (5′GGGTGTTTTTCCGAGTCAC 3′) or pd(N)6 random hexamer primers and reverse-transcribed at the recommended temperature using Superscript III reverse transcriptase (Invitrogen) or QuantiTect Reverse Transcription Kit (Qiagen), respectively. The reaction products were purified from the RNA by digestion with RNase H. Mandal 2009 cDNA was prepared as previously described [15].

### Nested PCR, sequencing, and phylogenetic analysis

Seven overlapping fragments were amplified by the nested PCR to obtain complete genomic sequences of Saringe 2009 and Mandal 2009. Primer sets were designed based on the complete genomic sequence of the Toro 2003 strain of the TBEV (GenBank Accession no. DQ401140.3). Nucleotide sequences and positions of the primers are listed in Table 2. KOD Hot Start Master Mix (Novagen) was used to amplify 2 µL of each cDNA using 0.3 µM of each set of primers and the PCR conditions described in Table 2. The PCR products were gel purified using Wizard SV Gel and PCR Clean-Up System (Promega) followed by sequencing (Eurofins MWG Operon, Ebersbeg, Germany). Genomic sequences of the Saringe 2009 and Mandal 2009 were deposited in the NCBI GenBank (KF991106 and KF991107, respectively). Nucleotide sequences were analyzed using the BioEdit version 7.1.3.0, Tom Hall Ibis Therapeutics, Carlsbad, CA). Phylogenetic analysis was performed with the MEGA5 software using the maximum-likelihood criteria [16].

**Table 2:** Primers and PCR conditions used for amplification of seven overlapping fragments (5′NCR-prM, E, E-NS3, NS3, NS3-NS5, NS4B-NS5, and NS5-3′NCR) to obtain complete genomic sequence.

PCR	Primer	Primer sequence (5′ 3′)	TBEV region (nt)	PCR conditions
5′NCR-prM	OF	AGATTTTCTTGCACGTGC	1–18 (+ sense)	35 cycles of 94°C, 15 s; 54°C, 15 s; and 70°C, 25
	OR	CCACCCAGTTCCAGCACCAA	1058–1039 (- sense)	
	IF	GTGCGTGCGTTTGCTTCGGA	15–34 (+ sense)	35 cycles of 94°C, 15 s; 54°C, 15 s; and 70°C, 25 s
	IR	GAGTACCAGTCACAAAGTCC	1018–999 (- sense)	
E	OF	CGGGGAGGGGACACAAATGG	785–804 (+ sense)	35 cycles of 94°C, 15 s; 52°C, 15 s; and 70°C, 30 s
	OR	AGGCATAGTTGTCATACC	2560–2543 (- sense)	
	IF	GTTGTGCTCCTGTGTTTGGC	940–959 (+ sense)	35 cycles of 94°C, 15 s; 56°C, 15 s; and 70°C, 30 s
	IR	CTCCATTCGTTCCGTGTC	2496–2479 (- sense)	
E-NS3	OF (Mandal 2009)	AGAAAGATTGACAGTGATAGGAGAGC	2202–2227 (+ sense)	35 cycles of 94°C, 15 s; 56°C, 15 s; and 70°C, 60 s
	OF (Saringe 2009)	CTGACAGTGATAGGAGAGCACGC	2209–2231 (+ sense)	
	OR	GCTGCTGACGTAGGTCTCATTAG	5097–5075 (- sense)	
	IF	TGCCTTCAACAGCATCTTCGG	2301–2321 (+ sense)	35 cycles of 94°C, 15 s; 56°C, 15 s; and 70°C, 60 s
	IR	CCCCACAACCACTCCCTG	5052–5035 (- sense)	
NS3	OF	CGTGACGAGAGGAGCGGCG	4761–4779 (+ sense)	35 cycles of 94°C, 15 s; 68°C, 15 s; and 70°C, 30 s
	OR	TGCGACGTGCCATGCCAG	6258–6241 (- sense)	
	IF	CTACTGGGCTGATGTGAGG	4809–4827 (+ sense)	35 cycles of 94°C, 15 s; 52°C, 15 s; and 70°C, 30 s
	IR	GTGAGTCGATAGTGACCGG	6182–6164 (- sense)	
NS3-NS5	OF	CTGACTTTGTGGTGACGACT	5822–5841 (+ sense)	35 cycles of 94°C, 15 s; 57°C, 15 s; and 70°C, 40 s
	OR	TATGCCCTGACACTCATGACTG	7973–7952 (- sense)	
	IF	GATGACAGTGGATTAGTGCAATGG	6046–6069 (+ sense)	35 cycles of 94°C, 15 s; 58°C, 15s ; and 70°C, 40 s
	IR	CCTTGAGGGTGGCATATCCGC	7888–7868 (- sense)	
NS4B-NS5	OF	ATGAGTGGCGTGGTCAGGGG	7582–7601 (+ sense)	35 cycles of 94°C, 15 s; 58°C, 15 s; and 70°C, 50 s
	OR	TCTCGCCGGTGAAAGTAGCTCAGC	9980–9957 (- sense)	
	IF	AAGCCTGTGGGGGTTCCTGCC	7602–7622 (+ sense)	35 cycles of 94°C, 15 s; 54°C, 15 s; and 70°C, 50 s
	IR	GCGTCCGTCCTTCATCACTA	9834–9815 (- sense)	
NS5-3′NCR	OF	ACACCCTCACCAACATAAA	9500–9518 (+ sense)	25 cycles of 95°C, 20 s; 55°C, 15 s; and 68°C, 37 s
	OR	GGGTGTTTTTCCGAGTCAC	11138–11120 (- sense)	
	IF	CTAGTGATGAAGGACGGACG	9814–9833 (+ sense)	25 cycles of 95°C, 20 s; 55°C, 15 s; and 68°C, 37 s
	IR	CACACATCACCTCCTTGT	11122–11105 (- sense)	

All the primers – outer forward (OF), outer reverse (OR), inner forward (IF), and inner reverse (IR) were identical for Saringe 2009 - and Mandal 2009 except E-NS3 OF. The nucleotide positions (nt) are related to the strain Toro 2003.

### 3′ NCR analyses

The NS5-3′NCR fragments of the Saringe 2009, Mandal 2009, 1991/4944, 1993/783, Habo 2011, Neudoerfl, Hypr, and Toro 2003 were amplified with the nested PCR (Table 2) and cloned into pcDNA3.1/V5-His-TOPO (Invitrogen) according to the manufacturer's instructions. Thirty different clones per strain were sequenced (Eurofins MWG Operon, Ebersberg, Germany) and the generated sequences were deposited in the NCBI GenBank (Saringe 2009 V1–V17 (KF991108, KF991114-KF991129), 1991/4944 (KF991110), 1993/783 (KF991109), Habo 2011 (KF991111), Neudoerfl V1-V2 (KJ960230-KJ960231).

### *In vitro* transcription

A control RNA for the V3′NCR was generated from already established TBEV DNA replicons based on the Toro 2003 sequence [17], [18]. *In vitro* transcription was performed using the MEGAscript SP6 Kit (Ambion). The components were mixed at room temperature according to the manufacturer's instructions, followed by 4 h incubation at 37°C. Replicon DNA was removed by digestion with TURBO DNase at 37°C for 15 min. RNA precipitation was performed using the lithium chloride method as per the manufacturer's instructions (Ambion).

## Results

### Sampling and sequencing of TBEV genomes from Norway and Sweden

In this study we generated genomic sequences of the TBEV from ticks sampled from two different sites in Scandinavia. Both sequences were generated directly from the total RNA extracts, but the virus was sampled at different stages within the life cycle of the tick. Mandal 2009 was sequenced from a pool of 10 questing *I. ricinus* nymphs sampled in southern Norway (58°0.43' N, 7°30' E) [15], whereas Saringe 2009 was sequenced from a single engorged nymph that had been blood-feeding for >60 h on a human male (collected in the northern region of the Stockholm area) (60°0.38′ N, 18°3.3′ E) [19]. The genomes were generated by traditional nested PCR amplification, and the overlapping products were sequenced directly to generate consensus genomes.

### The TBEV strains of Saringe 2009 and Mandal 2009 have different genomic organization of the V3′NCR

**Figure 1:**
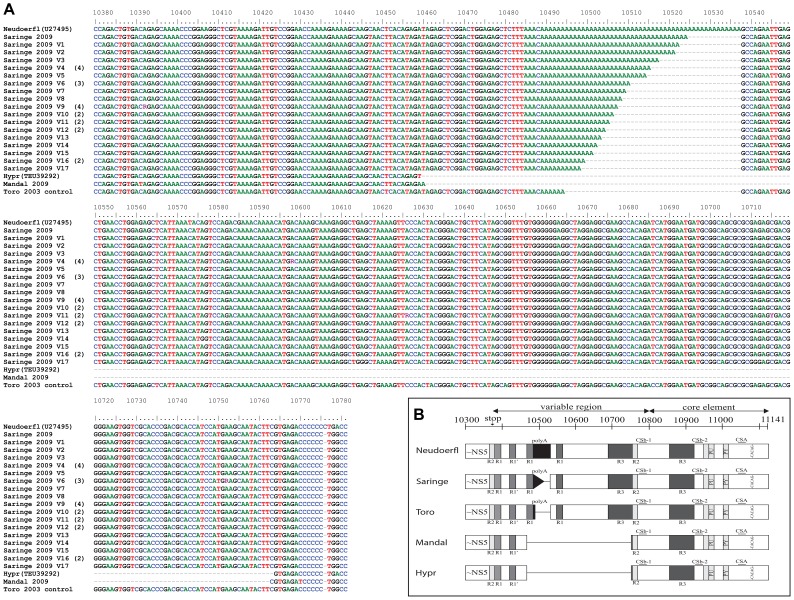
Genomic variations detected within the 3′NCR of the TBEV strains Saringe 2009 and Mandal 2009. (A) Alignment of 3′NCR partial sequences from pcDNA3.1 clones of the Saringe 2009 virus together with Neudoerfl, Hypr, Mandal 2009, and the Toro 2003 replicon used as control. Nucleotides' positions refer to the strain Neudoerfl. Different quasispecies variants of Saringe 2009 are labeled V1–V17 and number of clones with identical sequences is provided within parenthesis. (B) Schematic presentation of the 3′NCRs of Neudoerfl, Saringe 2009, Toro 2003, Mandal 2009, and Hypr showing heterogeneity in the V 3′NCR. The unique sequence elements: cyclization motifs (CSA, CSb-1, and CSb-2), repetitive sequences (R1, R2, and R3), poly(A) tract, homopurine box (PU), homopyrimidine box (PY), and flavivirus-conserved CACAG box are highlighted. Figure is adapted from Wallner et al., (1995).

The Saringe 2009 (Sweden) and Mandal 2009 (Norway) viruses were classified as W-TBEV (Figure S1), and the major parts of the genomes were highly conserved; however, significant differences between the two genomes were found within the V 3′NCR. Mandal 2009 was a short form of TBEV containing a 3′NCR with 464-nt. During the sequencing of the Saringe 2009, the peak height in both directions of the sequencing reactions dropped gradually and disappeared over the poly(A) region, which suggested fragments with different lengths. To resolve this sequence, products of the NS5-3′NCR PCR were cloned into the pcDNA3.1 vector and 30 clones of Saringe 2009 were separately sequenced, which revealed that the variability was due to the differences in the poly(A) tract. To assess the heterogeneity of the Saringe 2009 and Mandal 2009, the V 3′NCR sequences were aligned with prototypic W-TBEV strains, Neudoerfl and Hypr (Figure 1A). Interestingly, the Saringe 2009 contained a highly variable poly(A) region with fragments ranging from 10- to 36-nt long poly(A) (Figure 1A and B). Toro 2003 replicon control RNA was re-amplified, cloned and sequenced with the same procedure however, no elongation of the poly(A) tract could be detected in 30 clones (Figure 1A). The complete 3′NCR of Saringe 2009 was estimated to contain viral quasispecies with sequences varying from 725-nt - to 751-nt. The Mandal 2009 virus revealed a different genomic structure, as a large part of the V 3′NCR including the complete poly(A) region was absent (Figure 1A). Interestingly, the alignment shows that it is similar to the highly pathogenic Hypr strain (Figure 1A and B).

### Sequencing and characterization of V3′NCR of other W-TBEV strains

**Figure 2:**
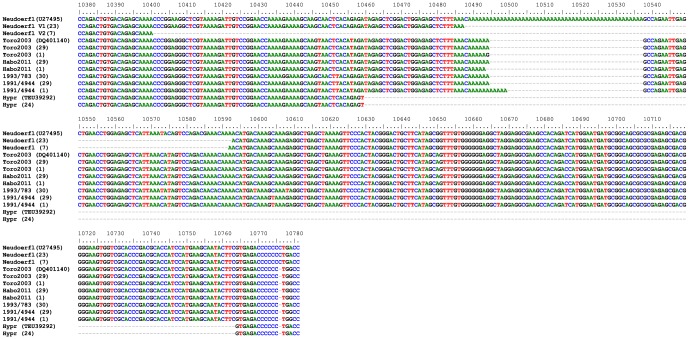
Genomic variations detected within the 3′NCR of W-TBEV strains. Alignment of 3′NCR partial sequences from pcDNA3.1 clones of the tick and human isolates of the TBEV to explore the existence of different variants within the individual viral pool. The two quasispecies variants of the Neudoerfl are labeled V1 and V2, respectively. Number of sequenced clones with identical sequences is mentioned within parenthesis. Nucleotide numbers correspond to the TBEV strain, Neudoerfl.

To investigate the putative presence of quasispecies within the additional TBEV strains, PCR products of NS5-3′NCR of strains Neudoerfl, Toro 2003, Habo 2011, 1993/783, 1991/4944, and Hypr were cloned and sequenced using the same procedure as for the Saringe 2009. Toro 2003 and Habo 2011 were amplified from the tick total RNA where Toro 2003 came from a questing tick pool [20] and Habo 2011 from a 20 h blood-fed nymph [19]. However, comparisons of 30 clones of each virus, respectively, only detected minor differences within the sequence populations (Figure 2). Thirty clones of the human TBEV isolates 1993/783 and 29 clones of 1991/4944 had a 6-nt long poly(A) region similar to Toro 2003; however, one clone of 1991/4944 contained an 11-nt long poly(A) (Figure 2). To our surprise the re-sequenced Neudoerfl genome completely lacked the poly(A) tract as 23 sequences had a deletion of nucleotide 10488–10592 and 7 sequences lacked nucleotide 10401–10591 (Figure 2). Nevertheless, the residual NS5-3′NCR sequence contained 35 Neudoerfl specific nucleotide markers and was identical to the parental strain published in the database (U27495) (data not shown). As expected, the Hypr sequence lacked large parts of the 3′NCR including the poly(A) region and contained no indications of detectable quasispecies among the sequenced clones (Figure 2).

### Phylogenetic relationship between the characterized viruses

**Figure 3:**
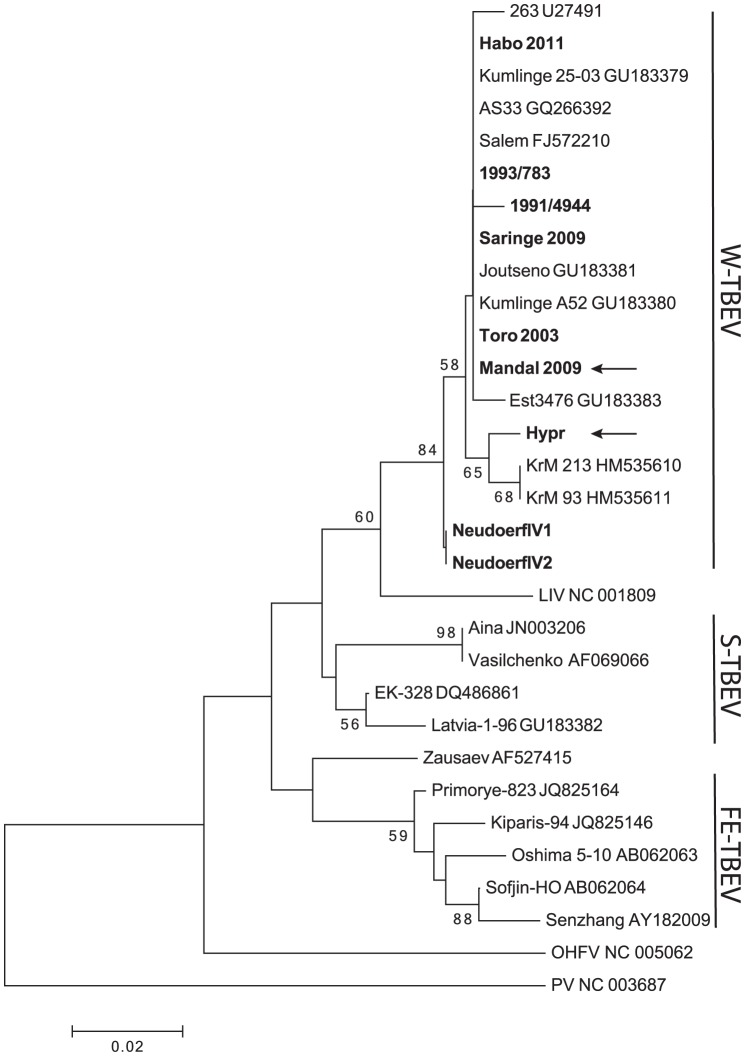
Phylogenetic analysis based on the 3′end of the NS5 gene (543 nt). Nucleotide sequences of 31 strains were analyzed by the Neighbor-joining method. The tree was inferred from 500 bootstrap replicates in MEGA5 [16], and the percentage of replicate trees is shown next to the branches [32]. The PV was used as outgroup and the tree was drawn to scale, with branch lengths corresponding to the substitutions per site. The TBEV strains sequenced in this study are indicated in bold. The positions of Mandal 2009 and Hypr are indicated by arrows.

The observed V3′NCR heterogeneity directed us to further characterize the phylogenetic relationship of these viruses. The sequence encoding the C-terminal region of NS5 was used to construct a phylogenetic tree (Figure 3). The sequenced viruses were all W-TBEV, but to get a representative tree for the mammalian tick-borne flavivirus group, we also included the sequence of additional W-TBEV, S-TBEV and FE-TBEV strains and one sequence of Louping ill virus (LIV), Omsk hemorrhagic fever virus (OHFV) and Powassan virus (PV), respectively. As expected, three separate clusters containing the W-TBEV, S-TBEV, and FE-TBEV appeared where the LIV could be placed within the W-TBEV cluster, and OHFV and the PV are more distantly related viruses (Figure 3). The W-TBEV cluster could be subdivided into clades, where Neudoerfl and Hypr appears to be separated from the clade including the Scandinavian TBEV strains. Interestingly, the short variants of W-TBEV i.e., Hypr and Mandal 2009 were localized in separate clades (Figure 3).

## Discussion

TBE infections in Sweden were first described in the year 1954 [21], and the disease has gradually been increasing and there are now up to 200–300 cases reported annually [5]. The first TBE case in Norway was described as late as 1997 [22], but the number of human infections has increased and in 2011 15 cases were reported. (Norwegian Institute of Public Health). To improve the understanding of the phylogeographic distribution and evolution of the TBEV, there is a persistent need for additional virus sequences. In this study, we sequenced and characterized two Scandinavian viruses directly from tick collections: Saringe 2009 and Mandal 2009, where the latter represents the first reported TBEV genome from Norway.

Generally, arboviruses evolve more slowly than other viruses as they are genetically constrained by selection through both vertebrate and invertebrate hosts. Traditionally, the E gene of the TBEV has been used in a number of phylogenetic studies [20], [23], [24], even though this gene is extremely conserved especially between the W-TBEV. One way of improving the phylogenetic resolution is to sequence other genes or full TBEV genomes [25], [26]. Phylogenetic analysis of the sequence encoding the complete polyprotein of TBEV placed the Saringe 2009 and Mandal 2009 in the W-TBEV clade and they sub-clustered with the previously published Toro 2003 sequence, indicating a Scandinavian TBEV cluster. Notably, Saringe 2009 is more closely related to the Toro 2003 than Mandal 2009 virus, which supports a closer geographical relationship between the two viruses detected within the Stockholm archipelago. Similar correlation between geographical and phylogenetic clustering has previously been reported for the TBEV in Slovenia based on partial gene sequences of the E protein and NS5 [27].

It has been suggested that the TBEV exists as a quasispecies population of mixed variants both within ticks and mammals, and the host switch may result in a rearrangement leading to the selection of the best-fit variants from the quasispecies population [13], [28]. However, the existence of the functional quasispecies within the natural life cycles of the RNA viruses have been challenged, which calls for additional studies in natural virus environments [29]. In this study, we show that the TBEV quasispecies exist in nature, as Saringe 2009 contains a highly heterogenic poly(A) tract ranging from (A)3C(A)10 to (A)3C(A)36. Saringe 2009 is sequenced directly from the total RNA of a single engorged tick, and we speculate that the observed heterogeneity might be due to the rearrangements within the quasispecies pool favored by the mixed environment of the tick cells in the presence of mammalian blood at 37°C observed after more than 60 h of blood-feeding. Habo 2011 had a similar host background, but the host tick had only been feeding for 20 h. The Habo 2011 virus contained homogenously (A)3C(A)6 clones similar to Toro 2003, which originates from the questing ticks. This indicates that the quasispecies rearrangement either requires longer blood-feeding periods or that the pool of present quasispecies is different for the Habo 2011 virus. 1993/783 and 1991/4944 are two isolates from the Swedish TBEV patients [14], and all clones of 1993/783 and 29 out of 30 clones of 1991/4944 contained homogenic (A)3C(A)6 poly(A) sequences, suggesting this to be a dominant genomic structure. Interestingly, one clone of 1991/4944 contained an (A)3C(A)11 sequence, indicating that the quasispecies of TBEV with longer poly(A) tracts may also be present in the virus isolated from human patients.

Two European TBEV control strains, Neudoerfl and Hypr, were processed here with the same strategy of amplification followed by cloning and sequencing. Neudoerfl has previously been reported to contain an internal poly(A) region, however our re-sequenced strain surprisingly revealed two distinct quasispecies populations, both lacking the poly(A) region. Laboratory propagation has previously been shown to have manipulating effects on the V3′NCR in Neudoerfl, as when either the virus or an infectious Neudoerfl clone was passaged in the suckling mice brain and/or propagated in mammalian cells, truncated virus variants within the V 3′NCR evolved [12]. We do not have the exact passage history of the isolate used herein; however, several nucleotide markers showed that the virus is Neudoerfl. We believe that the two detected quasispecies variants are due to truncations, which occurred during passaging in the laboratory, rather than having specific roles in the natural life cycle of the Neudoerfl virus.

It was previously demonstrated that the complete V 3′NCR could be removed without effect on the virulence of Neudoerfl [12]. The fact that the highly virulent Hypr strain is an old isolate, lacking most parts of the V 3′NCR, have led to speculations that this virus has evolved by truncations and adapted to the laboratory environments during repeated cultivation in mammalian cells [30]. In our study, we show that the Mandal 2009 virus contains a homogenous V 3′NCR similar to the Hypr strain, which could be an important virulence determinant of the Norwegian strain. We have previously suggested that the TBEV genome of Toro 2003, with the (A)3C(A)6 poly(A) tract is the typical W-TBEV detected within the questing ticks, as that sequence came directly from the tick total RNA [30]; however, here we demonstrate that the shorter TBEV variants indeed also exist in nature within the questing ticks. The presence of the short TBEV variants in Scandinavia could either indicate that the virus has spread to Norway from eastern parts of Europe or that it has arisen due to the genomic instability of the TBEV within the V3′NCR during the natural life cycle. Based on the phylogenetic trees, the coding part of the virus seems to be more closely related to the Swedish W-TBEV rather than Hypr, which suggests that Mandal 2009 has evolved by truncation of longer Scandinavian TBEV variants.

Interestingly, a recent study on FE-TBEV indeed highlighted the V 3′NCR as a highly significant virulence factor of TBEV [31]. Here, we have studied the TBEV in Sweden and Norway and showed that short V 3′NCR TBEV, similar to the Hypr, exists in the Norwegian questing ticks. We also investigated the heterogeneity in the 3′NCR of TBEVs from Swedish TBE patients and blood-feeding ticks. Altogether, the study highlights the variation in the TBEV genome at different life stages and at different collection sites in Scandinavia, with putative connections to differences in the virulence. Our findings further demonstrate the existence of diverse variants of TBEV within the viral pool of a blood-fed tick. We assume that the viral pool is quite dynamic and certain parameters like prolonged blood-feeding may instigate the rearrangement of the quasispecies pool. We speculate that at a certain time point during the blood meal the TBEV population in the tick shifts for optimal transmission to the vertebrate and a new balance emerges in the quasispecies pool. Our new data highlights the importance of additional studies on V 3′NCR and the poly(A) tract using different strains and infectious clone model to elucidate its role in TBEV virulence and vector to vertebrate transmission.

## Supporting Information

Figure S1 **Phylogenetic analysis based on open reading frame sequences of TBEV.** Nucleotide sequences of 90 strains were analyzed by the Maximum Likelihood (ML) method with a total of 10237 positions in the final dataset. The evolutionary history was tested by using the ML method based on the GTR +G+I as the best-fit substitution model. The bootstrap confidence is based on 500 replicates of the ML analysis. Omsk sequences were used as outgroup. Evolutionary analysis was conducted in MEGA5 and the Genbank accession numbers are indicated.
(EPS)
